# Modifying PTEN recruitment promotes neuron survival, regeneration, and functional recovery after CNS injury

**DOI:** 10.1038/s41419-019-1802-z

**Published:** 2019-07-29

**Authors:** Alireza Pirsaraei Shabanzadeh, Philippe Matteo D’Onofrio, Mark Magharious, Kyung An Brian Choi, Philippe Patrick Monnier, Paulo Dieter Koeberle

**Affiliations:** 1 Division of Anatomy, Department of Surgery, University of Toronto, Toronto, ON M5S 1A8 Canada; 2 Departments of Physiology, University of Toronto, Toronto, ON M5S 1A8 Canada; 3 Rehabilitation Science Institute, University of Toronto, Toronto, ON M5S 1A8 Canada; 4 Krembil Research Institute, University Health Network, Toronto, ON M5T 2S8 Canada

**Keywords:** Stroke, Trauma

## Abstract

Phosphatase and tensin homolog (PTEN) regulates apoptosis and axonal growth in the developing and adult central nervous system (CNS). Here, we show that human PTEN C-terminal PDZ interactions play a critical role in neuronal apoptosis and axon regeneration after traumatic CNS injury and stroke, highlighted by the findings that antagonizing the PDZ-motif interactions of PTEN has therapeutic applicability for these indications. Interestingly, the death-inducing function of PTEN following ischemic insult depends on a PDZ-domain interaction with MAGI-2 and MAST205, PDZ proteins that are known to recruit PTEN to the plasma membrane and stabilize its interaction with PIP3. Treatments with a human peptide that prevents PTEN association with MAGI-2 or MAST205 increased neuronal survival in multiple stroke models, in vitro. A pro-survival effect was also observed in models of retinal ischemia, optic nerve transection, and after middle cerebral artery occlusion (MCAO) in adult rats. The human PTEN peptide also improved axonal regeneration in the crushed optic nerve. Furthermore, human PTEN peptide therapy promoted functional improvement after MCAO or retinal ischemia induced via ophthalmic artery ligation. These findings show that the human peptide-based targeting of C-terminal PTEN PDZ interactions has therapeutic potential for insults of the CNS, including trauma and stroke.

## Introduction

Adult mammalian central nervous system (CNS) neurons have limited regenerative capacity and are highly susceptible to apoptosis (programmed cell death) following injury^1,2^. Factors of the CNS microenvironment contribute to this effect^3,4^, including the absence of Schwann cells^1^, and the accumulation of growth-inhibiting compounds at the injury site^5,6^. Inhibition of these antagonistic factors greatly improves axonal regeneration^7^, indicating that modification of the downstream signals resulting from environmental contact presents a promising avenue for the enhancement of neuron survival and regeneration.

Ischemic CNS injury, encompassing brain and retinal ischemia, presents a significant health concern worldwide. In the United States, the prevalence of stroke is roughly 3% of the adult population^8^. Meanwhile, retinal ischemia is a prominent cause of visual impairment and blindness worldwide^9^, and shares many similarities with cerebral ischemia: the retina is an extension of the diencephalon, so retinal blood vessels share similar anatomical, physiological, and embryological properties, possessing a blood–retinal barrier analogous to the blood–brain barrier^10^. As such, ischemic injury to the CNS represents a critical unmet need for therapeutic intervention.

CNS ischemia causes neuronal energy depletion and apoptosis^11,12^. Thromboembolic stroke models are valuable in studying cerebral ischemic infarction, because they recapitulate the hallmark symptoms of human cerebrovascular disease^13–15^. The retina and optic nerve also form a system that is well suited for CNS experimentation^16^, as the optic nerve can be reproducibly injured to disrupt the axons of all the retinal ganglion cells (RGCs)^17^. Retinal ischemia, induced by ligation of the ophthalmic artery, is a reproducible model of CNS stroke that progresses in a predictable manner: ~50% of RGCs die within the first 2 weeks after stroke^18^. This model is also highly amenable to experimental manipulations^9,19^, as it is more easily accessible than the brain. This study used a combination of both injury models to address the question of how modifying phosphatase and tensin homolog deleted from chromosome 10 (PTEN) C-terminal PDZ interactions can affect the outcome of traumatic or ischemic injury.

PTEN indirectly inhibits PKB/Akt activity by interfering with PIP3 accumulation. PTEN negatively regulates the intracellular levels of PIP3 in cells and dephosphorylates PIP3 that is required for PI3K function^2^. This, in turn, antagonizes PI3K/AKT/mTOR pro-survival signaling^20^. Several studies have shown that Akt signaling is necessary for neuron survival following trophic factor deprivation, oxidative stress, and ischemic injury^11^. Furthermore, alterations of the levels of PTEN have been reported in a rat model of transient cerebral ischemia, particularly in damaged brain regions^12^. Moreover, several studies have shown that neuronal survival can be enhanced by the downregulation of PTEN activity^21,22^.

Exercise can increase neuronal proliferation, survival, and net neurogenesis in the CNS^23^, concomitant with an increase in brain-derived neurotrophic factor (BDNF) levels^24^, as observed in the hippocampus and cerebral cortex^25^, and in the spinal cord^26^. Furthermore, exercise increases the levels of protein kinase A^24^, which is involved in the BDNF signaling, and contributes to overcoming the inhibitory effects of myelin on regeneration^27^. BDNF supports the survival of neurons and activates both the MAPK and PI3K pathways in the retina following optic nerve axotomy^28^. These pathways are also activated by exercise^29^, suggesting that exercise may be useful in modulating neuron survival following CNS injury.

PDZ (PSD95/Dlg1/ZO-1 homology) protein-interacting domains are the most common type of protein–protein interaction domains and act as specific “pockets” for PDZ-binding motifs^30,31^. PDZ motifs are usually four residues long, where amino acid 0 (the C-terminal residue) and residue −2 are the most important for recognition by the PDZ domain^31^. Peptides that replicate PDZ-binding motifs can be synthesized to antagonize specific PDZ interactions^30^.

The present study tested if the activity of the PI3K or MAPK pathways could be modulated by forced exercise to prevent CNS neuron apoptosis. An alternative approach via targeting the PDF-binding motif of PTEN phosphatase to achieve the same effect, was also tested, revealing potent neuroprotective effects in the visual system and cerebrum of the adult mammalian CNS.

## Materials and methods

### Treadmill exercise

We assessed the effects of treadmill training on RGC survival and intracellular messenger activation after injury. A total of 18 animals were randomly used: 6 were used to evaluate the effects of exercise on the activation of the PI3K and MAPK pathways; 12 were used to evaluate the role of exercise in preventing RGC death after axotomy. All animals were run on an Exer3-lane treadmill (Columbus Instruments, Columbus, OH).

The group of six animals was randomly divided into light-exercise and heavy-exercise subgroups. Light-exercise animals underwent a 3-week (5 days/week) exercise program in which animals ran at 15.0 m/min for varying durations, starting at 10 min/day and increasing to 40 min/day; this represents a total daily distance between 150 and 600 m. Heavy-exercise animals underwent a 3-week (5 days/week) exercise program, running at 22.0–25.0 m/min for 60 min/day; this represents a total daily distance of 1320–1500 m. Animals were killed on the final day of training, and their tissue was processed for western blotting. These samples were used to assess the levels of Akt (Antibody#9272, Cell Signaling Technology, ON, Canada), phosphorylated Akt (Antibody#9916, Cell Signaling Technology, ON, Canada), MAPK (Antibody#9926, Cell Signaling Technology, ON, Canada), and phosphorylated MAPK (Antibody#4511, Cell Signaling Technology, ON, Canada) in exercised and normal adult rats.

To test for the effects of exercise on CNS neuron survival after injury, 12 animals underwent a 2-week (5 days/week) exercise program, running at 20.0 m/min for 20–60 min/day. This represents a total daily distance of 400–1200 m. Optic nerve transection was performed on the final day of the exercise program, and RGC survival was assessed at 7 days (*n* = 6) or 14 days (*n* = 6), and compared with non-exercised animals.

### Western blots

Activation of the PI3K and MAPK pathways following different treatments was assessed via western blot on the whole retina tissue. Animals were killed, their eyes enucleated, and the retinas immediately extracted in ice-cold PBS. Retinas were individually sonicated in 400-μl solutions of ice-cold SDS lysis buffer (2% SDS, 0.3% DTT, and 10% glycerol in 40-mm Tris-Cl, pH 6.8) prior to denaturing (heated to 90 °C for 8 min) and removal of cellular debris by centrifugation (12,000 rpm, 10 min, 4 °C).

Proteins were separated by SDS-PAGE on 10% acrylamide Bio-Rad TGX gels and then transferred via semidry blotting to a 0.2 -μm pore nitrocellulose membrane. Membranes were blocked with 5% milk in TBS-T for 1 h at room temperature. Primary antibody incubation occurred in 1% milk in TBS-T overnight at 4 °C on a platform rocker. The secondary antibody was HRP-conjugated, and incubation was performed for 1 h at room temperature in 5% milk in TBS-T, on a platform rocker.

Blots were imaged using a Bio-Rad Fluor-S Max imager (Bio-Rad, Mississauga, ON). Loading was verified by re-probing the blots with antisera directed against GAPDH (1:1000; rabbit polyclonal; Cell Signaling Technology). Normalized densitometry values (relative to GAPDH) for each experimental group were averaged ±SEM, and statistical analysis was performed by ANOVA followed by post hoc analysis using Tukey’s post hoc comparisons.

### Optic nerve transection and retinal ischemia

Adult female Sprague Dawley rats, free of common pathogens, were used in all experiments. Optic nerve transection or optic nerve crush were performed as we have previously described^32–34^. Animals were placed in a stereotaxic frame and anesthetized with isoflurane (2%; 0.8 L/min oxygen flow rate) delivered through a gas anesthesia mask. The optic nerve was accessed within the ocular orbit via an incision in the tissue covering the superior border of the orbital bone. The dural sheath surrounding the optic nerve was cut longitudinally, to avoid damaging blood vessels supplying the retina. The optic nerve was gently lifted from the meningeal sheath and transected within 2 mm of the back of the eye. In order to retrogradely label RGCs, gelfoam soaked in 2% Fluorogold (Sigma) was placed over the transected optic nerve stump. The optic nerve crush procedure was carried out using a similar approach, but the optic nerve, with meningeal coverings intact, was crushed for 6 s using fine self-closing forceps.

The approach for temporary retinal ischemia (ophthalmic artery ligation) was similar, with a few exceptions^35^. Since the optic nerve was not damaged in this procedure, RGCs had to be retrogradely labeled prior to ischemia. One week prior to surgery, animals received stereotaxic injections of 2% Fluorogold into the CNS target of RGC, the superior colliculus. The surgical approach to the optic nerve was identical to that described for optic nerve transection. After a longitudinal cut was made in the meningeal sheath surrounding the optic nerve, the nerve was gently lifted out of the sheath. The meningeal sheath, including the ophthalmic artery, was then clamped using a calibrated microvessel clamp for 45 min, after which the clamp was removed to permit vascular reperfusion. The reduction in retinal blood flow and postsurgical reperfusion was verified by fundus examination.

### Intraocular injections, optic nerve injections, I.V. injections, and I.P. injections

We have previously described the procedures for intraocular (IO) and optic nerve injections^32–34,36^. Animals received IO injections of each peptide at 3 and 8 days post axotomy, prior to the onset of RGC apoptosis, which occurs at 4–5 days after axotomy. Animals were placed in a stereotaxic frame and anesthetized with isoflurane, delivered through a gas anesthetic mask. The cornea was anesthetized using Alcaine eye drops (Alcon) prior to IO injections. A pulled glass micropipette, attached to a 10-µl Hamilton syringe via a hydraulic coupling through PEEK tubing, was used to deliver 4 µl of a peptide solution (10 mg/ml) into the vitreous chamber of the eye, posterior to the limbus. Care was taken to prevent damage to the lens or anterior structures of the eye. The pipette was held in place for 5 s after injection and slowly withdrawn from the eye to prevent reflux. Injections were performed using a surgical microscope in order to visualize pipette entry into the vitreous chamber and confirm delivery of the injected solution.

PTEN PDZ interactions were antagonized using cell-permeable peptides based on the PDZ-binding motif of PTEN (Table 1). The protein transduction domains consisted of a fragment of the HIV TAT protein (Y-G-R-K-K-R-R-Q-R-R-R^37–39^; or a sequence of nine arginine residues (R9))^40–43^. Both PDZ peptides were dissolved in normal saline and delivered via IO injection at a concentration of 10 mg/mL (4 µl intraocular) at 3 days following optic nerve transection or ophthalmic artery ligation.

**Table 1:** PDZ peptides

PDZ peptide	Transduction n component	Sequence of active component	Source of active component
TAT-PTEN	TAT	D-Q-H-S-Q-I-T-K-V	C-terminal residues of PTEN protein
R9-PTEN	R9	D-Q-H-S-Q-I-T-K-V	C-terminal residues of PTEN protein
P-peptide	P	D-Q-H-S-Q-I-T-K-V	C-terminal residues of PTEN protein
P-peptide—pT	pT	D-Q-H-S-Q-I[pT]-T-K-V	C-terminal residues of PTEN protein
HP peptide	HP	D-Q-H-T-Q-I-T-K-V	C-terminal residues of PTEN protein

The peptides consisted of two components covalently bound together: an active component composed of an amino acid sequence containing the PDZ-binding motif involved in the target PDZ interaction; and a protein transduction component to mediate the transportation of the peptide into the cell

To directly target RGCs, peptide solutions were injected into the transected optic nerve stump using a 10-μl Hamilton syringe. A total of 5 μl of peptide suspension (10 mg/ml) was injected. The majority of the injected fluid will reflux out of the optic nerve during this procedure, so the remaining pool of peptide suspension was left in place when returning the orbital contents to their original positions.

For intravenous injections, a bolus of 1 ml of saline containing 0.5 mg of dissolved peptide was delivered via a tail-vein injection at both 24 h and 8 days following ophthalmic artery ligation (stroke). Injections were delivered using a 25-gauge needle attached to a 5-ml syringe, while animals were anesthetized with isoflurane.

For intraperitoneal (IP) injections, 1 ml of saline containing 0.5 mg of dissolved peptide was delivered via a 25-gauge needle attached to a 5-ml syringe, following rapid anesthesia with isoflurane to incapacitate the animal.

To test the effect of PTEN on regeneration in the axotomized optic nerve, animals were randomized into four groups: a control group that received IO injections of normal saline (control vehicle, *n* = 8); a group that received treatment of TAT-PTEN via IO injection (*n* = 6); a group that received Gelfoam soaked in TAT-PTEN solution (*n* = 6), applied to the cut end of the optic nerve; a group that received R9-PTEN treatment via IO injection (*n* = 4). To test the effect of PTEN on RGC survival in the ischemic retina, animals were randomized into three groups (*n* = 7 each): a control group that received IO injections of normal saline (control vehicle) following surgery; a sham-surgery group that received a sham surgery (all steps were followed with the exception of the ligation step) and did not receive IO injections; a treatment group that received ligation surgery followed by IO injections of PTEN (R9-PTEN; Table 1).

### Quantification of RGC survival after injury

Eyes were enucleated, the cornea and lens were removed and the remaining eye cups containing the retinas were fixed in 4% paraformaldehyde (PAF) at 14 days post axotomy. Eye cups were fixed for 1 h and then rinsed in PBS for 15 min. The retinas were then extracted, flat-mounted and cover-slipped using 50:50 glycerol: PBS. Fluorogold staining in RGCs was visualized using an Andor iXon 885+ EMCCD camera attached to a Leica DM LFSA microscope. The illumination source was a Sutter Lambda XL with a liquid light guide ensuring even field illumination. RGC densities were sampled at the inner (1/6 retinal eccentricity), mid-periphery (1/2 retinal eccentricity), or outer retina (5/6 retinal eccentricity) of each quadrant of the flat mount. RGC densities were grouped by retinal eccentricity (inner, middle, and outer), and expressed as mean ± SEM. ANOVA followed by post hoc analysis, using Tukey’s post hoc comparisons (*p* < 0.05), was used to determine statistical significance between experimental and control samples.

### Quantification of RGC axon regeneration

Retinal ganglion cell axon regeneration was assessed at 21 days after optic nerve crush as previously described^36^. On day 21, animals were given an intracardial perfusion of PBS followed by 4% paraformaldehyde, and their optic nerves were carefully removed. Nerves were frozen-sectioned, and processed for immunohistochemistry directed against growth associated protein-43 (GAP-43; rabbit polyclonal, 1:250, Cell Signaling Technology/NEB), which is expressed in neuronal growth cones during development and axonal regeneration^44,45^, and is expressed by RGCs during axon outgrowth^46,47^. Four equally spaced sections were examined across the width of each optic nerve. These were imaged using a Leica DM LFSA microscope (Leica Microsystems, Concord, Canada) at 20× objective, with an Andor iXon 885 + camera (Andor Technology, Belfast, Northern Ireland), and with EM gain applied. The total number of regenerating axon growth cones was quantified based on distance from the crush site: 0–250 μm; 250–500 μm; >500 μm. The total number of regenerating axons at each distance was averaged and expressed as a mean ± SEM. Statistical analysis was performed by ANOVA and Tukey’s post hoc test.

### Cortical neuron culture and glutamate cytotoxicity

Cortical neuron cultures were made from newborn (P5) rat pups. Cortical samples were triturated 30× using a P1000 pipettor in cold Dubelcco’s PBS + glucose. Neurons were plated in 24-well plates coated with poly-d-lysine. A total of 100,000 cells/well were plated in cold Neurobasal A medium + glutamax + penicillin/streptomycin. Cultures were placed in an incubator (37 Celsius, 5% CO_2_) for 14 days, with half the media changed after 3 days.

After 14 days in culture, the media was replaced with fresh media containing 5 mM glutamate. Experimental samples received various amounts of each peptide, whereas controls received an equivalent volume of PBS (vehicle). After 24 h, cell survival was quantified using the crystal violet assay: Media was removed from the cells and 200 μl of 0.2% crystal violet (dissolved in 20% methanol: 80% H_2_O) was added to each well. After 5 min, wells were rinsed with distilled water and the plates were dried. Crystal violet was eluted from the cells using 0.1 M sodium citrate in 50% ethanol. A total of 100 μl of samples from each well were then transferred to 96-well plates and absorbance was read at 540 nm. The results were expressed as the relative fold change in comparison with the control samples that received buffer.

### Oxygen–glucose deprivation in vitro

HEK293 cells were seeded at a density of 40,000 cells/35-mm dish. OGD experiments were performed after 2 days of culture^48–50^. After three washouts with glucose free Earl’s balanced salt solution (BSS), cells were maintained in the same BSS medium, (140 NaCl, 5.4 KCl, 1.2 CaCl_2_, 0.9 MgCl_2_, 0.44 KH_2_PO_4_, 4.17 NaHCO_3_, and 0.34 Na_2_HPO_4_ in mM). Prior to use, BSS was equilibrated with the anaerobic gas mixture (95% CO_2_/3.8% N_2_/1.2 O_2_) by bubbling for 15 min, adjusted to pH 7.4 if necessary, and heated to 37 °C. Cells were then placed in a humidified incubator at 37 °C in anaerobic gas conditions for 2 h. After OGD, cells were washed with phosphate-buffered saline solution (PBS, Invitrogen, Cergy-Pontoise, France) and fixed with paraformaldehyde 4% at 4 °C. Cells were washed three times with PBS. Then, nuclei of living cells were labeled by Hoechst during 10 min at 4 °C. Cells were washed with PBS (X3) and labeled cells were visualized using a video microscope with a Metafluor software. Cells were counted automatically by ImageJ software and results were expressed as mean ± SEM. OGD was carried out on native HEK293 cells with three groups: control group (non-OGD); OGD alone, and R9-PTEN treatment.

Trypan blue dye passes into the cytoplasm of membrane-damaged cells, whereas undamaged cells exclude the dye. This property was used to study cell viability by assessing the loss of membrane integrity following the method of Pant et al. with desired modifications. Each batch of culture was assessed for cell viability at 1 day after OGD insult^51,52^. The MTT assay was used as an additional measure of cell viability after OGD. After peptide treatments, cells were incubated with MTT (1 mg/ml) for 4 h at 37 °C. Mitochondrial dehyrogenases of viable cells cleave the tetrazolium ring of the yellow MTT to yield purple formazan crystals which are insoluble in aqueous solutions. The crystals were dissolved in 100 μl of DMSO and the absorbance of the resulting purple solution was measured at 570 nm against 690 nm for blank solution. The amount of produced formazan is directly proportional to the number of viable cells^53^_._

### Elevated intraocular pressure

Adult female Long–Evans rats (body weight 160–180 g) were anesthetized with an isoflurane vaporizer. Each anterior chamber was cannulated with a 26-gauge infusion needle connected to a normal saline (0.9% sodium chloride) container through Silastic tubing (0.5-mm diameter, Dow Corning, USA)^54^. The IOP in the cannulated eyes was raised to 110 mm Hg for a period of 60 min by elevating the saline container. Total eye ischemia was evident due to whitening of the anterior segment of the eye and concurrent blanching of the retinal arteries in the fundus. The ischemic insult was ended by withdrawal of the needle from the anterior chamber. Vascular reperfusion was confirmed via fundoscopy and animals were placed under a heat lamp to recover.

Animals were randomized into two groups (control and treatment, *n* = 7 each) to examine the effects of PTEN on RGC survival following high IOP injury. Both groups received two IO injections following the high IOP injury: immediately after, and at 3 days after. The control group received injections of normal saline, and the treatment group received injections of R9-PTEN peptide.

Animals were killed at 7 days post axotomy: deep anesthesia was induced by sodium pentobarbital and animals were immediately perfused through the heart with Hanks’ solution (Nissui Pharmaceutical, Tokyo) followed by 4% glutaraldehyde in 0.1 mol/I sodium cacodylate buffer solution. Eyes and optic nerves were removed and postfixed in the same fixative at 4 °C overnight. RGC survival was quantified as described earlier.

### Functional assessment of visual acuity and optomotor response

The optomotor response was measured at 7 days after IOP elevation^55,56^. Rats were placed on a platform in the form of a grid (11.5-cm diameter, elevated to 19.0 cm) surrounded by a motorized drum (29.0-cm diameter) that could be revolved clockwise or anticlockwise at two revolutions per minute, the optimal velocity for evoking an optokinetic response in the mouse. After 10 min of adaptation in the dark, vertical black and white stripes of a defined spatial frequency were presented to the animal. These stripes were rotated alternately clockwise and anticlockwise, for 2 min in each direction with an interval of 30 s between the two rotations. Various spatial frequencies subtending 0.03, 0.13, 0.26, 0.52, and 1.25 cpd (cycles/degree) were tested individually in a random sequence. Animals were videotaped with a digital video camera (Sony, DCR-TRV24E) for subsequent scoring of head-tracking movements. Head movements were scored only if the angular speed of the head corresponded to that of the drum rotation. If the spatial frequency of the black and white stripes was increased, a threshold was reached beyond which no tracking movements of the head were detected. The visual spatial resolution (or visual acuity) of the animal was estimated to be greater than or equal to this threshold but below the next spatial frequency tested.

### Induction of thromboembolic focal cerebral ischemia

In order to examine the effects of the peptide therapeutics described herein, a thromboembolic model of middle cerebral artery (MCA) occlusion that closely mimics human ischemic brain stroke was used: a clotted segment of blood is injected into the origin of the MCA to block blood flow in the corresponding vascular territory of the lateral cerebrum^14^.

Following deep isoflurane anesthesia via a nose cone, fur was shaved from the ventral aspect of the neck. The shaved skin was cleaned twice with alternating applications of Proviodine (iodine detergent solution) and 70% ethanol. Following a final application of Proviodine, the surgical field was draped, and a midline incision made in the ventral skin and fascia of the neck. Under an operating microscope, the right common carotid artery (CCA), the right external carotid artery (ECA), and the right internal carotid artery (ICA) were carefully isolated and separated from the adjacent vagus nerve. A 6–0 silk suture was loosely tied at the origin of the ECA, and another suture was used to ligate the ECA distally. A sterile modified PE 10 catheter was introduced into the lumen of the right ECA via a small puncture. Ten microliters of blood was withdrawn into the catheter and retained for 15 min to allow formation of a clot. The fresh autologous preformed clot was used for MCA occlusion. The right CCA and the right ICA were temporarily clamped using microvascular clips. The PE 50 catheter containing a 20-mm-long fibrin-rich clot was attached to a 100-μl Hamilton syringe and introduced into the lumen of the ECA via a small incision. The suture around the origin of the ECA was tightened around the intraluminal catheter to prevent bleeding, and the microvascular clip on the ICA was removed. The catheter was gently advanced from the ECA into the lumen of the ICA and upward to the origin of the MCA (a total of 17 mm in distance from the bifurcation of the CCA). At this point, the end of the catheter is 1–2 mm away from the origin of the MCA. Over 10 s, the clot (3.5-μl volume) was gently injected together with 5 μl of sterile saline within the catheter. The catheter was removed 5 min after injection, and the right ECA was ligated at its origin. Body temperature was maintained at 37 °C with a heating pad for the duration of surgery, after which the surgical field was closed with 4–0 silk sutures. Subcutaneous injections of buprenorphine (0.05 mg/kg) were administered to minimize postsurgical discomfort.

### Intravenous injections of experimental peptides after MCAO

Experimental animals were anesthetized with isoflurane (via a gas mask attached to a stereotaxic frame) and received tail-vein injections of peptide solutions. Dissolved peptides were injected into the tail vein at a dose of 1 mg/kg, in a 0.5-ml volume. Control injections consisted of sterile vehicle.

The effects of hPTEN, PTEN, and pPTEN on ischemic brain injury in norm thermic rats were studied after MCAO. Animals were randomized into four groups as follows: control (DMSO, *n* = 7), hPTEN (*n* = 7), PTEN (*n* = 7), and pPTEN (*n* = 7). All treatments were dissolved in sterile DMSO. hPTEN, PTEN, and pPTEN were delivered at a dose of 1.5 mg/kg via intravenous (tail-vein) injection. Since the number of necrotic neurons increases most markedly during the 6–12-h interval after MCA occlusion^57^, and the time course of cortical apoptosis is <24 h after MCAO^58^, the initial peptide treatment was administered immediately after MCAO and repeated at 24 h in the present study.

### Quantification of brain infarct volume and edema

Forty-eight hours after MCA occlusion, isoflurane-anesthetized rats were euthanized by decapitation. The brains were removed from the skull and cooled in ice-cold saline for ~5 min. For morphometric examination, 2-mm-thick coronal sections were cut using a rat brain matrix. A total of eight coronal sections were collected, and the sections were stained using a 2% 2,3,5-triphenyltetrazolium chloride solution (TTC). TTC is metabolized by live cells, turning the viable regions of the brain a reddish-brown color; whereas dead regions of brain tissue remain white when viewed with the naked eye. Infarct volumes were calculated from scanned images of the coronal surfaces of brain slices, using the following formula: Infarct volume = (volume of uninjured hemisphere−(volume of injured hemisphere−measured infarct volume))/volume of uninjured hemisphere) × 100. This formula serves to quantify the percentage of the injured hemisphere that is infarcted (dead), relative to the uninjured hemisphere volume, in order to account for edema or differences in volumes between the two hemispheres. Brain edema was calculated by comparing the relative volume of the injured brain hemisphere to the uninjured hemisphere using the following formula: Edema % = ((volume of injured hemisphere−volume of uninjured hemisphere)/volume of uninjured hemisphere) × 100. This calculation yields a percentage value that represents an increase in volume of the injured hemisphere (due to edema) relative to the uninjured hemisphere. Four to six brains for each treatment group or controls were analyzed and the mean ± SEM of infarct volume % or brain edema % was calculated for each group. Statistical significance was determined using Tukey’s post hoc tests following an ANOVA at *p* < 0.05.

### Neurological deficits and seizure activity

Neurological deficits and seizure activity of each rat were evaluated at 2, 8, 24, and 48 h following ischemic injury by an observer who had no knowledge of which procedure had been performed. Neurological deficits and seizure activities were classified with Bederson’s and Racine’s scoring systems^59,60^.

Bederson’s scoring system: 0, no observable deficit (normal); 1, forelimb flexion (moderate); 2, forelimb flexion plus decreased resistance to lateral push (moderate); 3, unidirectional circling (severe); 4, unidirectional circling plus decreased level of consciousness (severe).

Racine’s scoring system: 0, no seizure activity (normal); 1, rhythmic mouth and facial movement (moderate); 2, rhythmic head nodding (moderate); 3, forelimb clonus (severe); 4, rearing and bilateral forelimb clonus (severe); 5, rearing and falling over (very severe)^59^.

## Results

### Effects of forced exercise on PI3K and MAPK activation

**Fig. 1:**
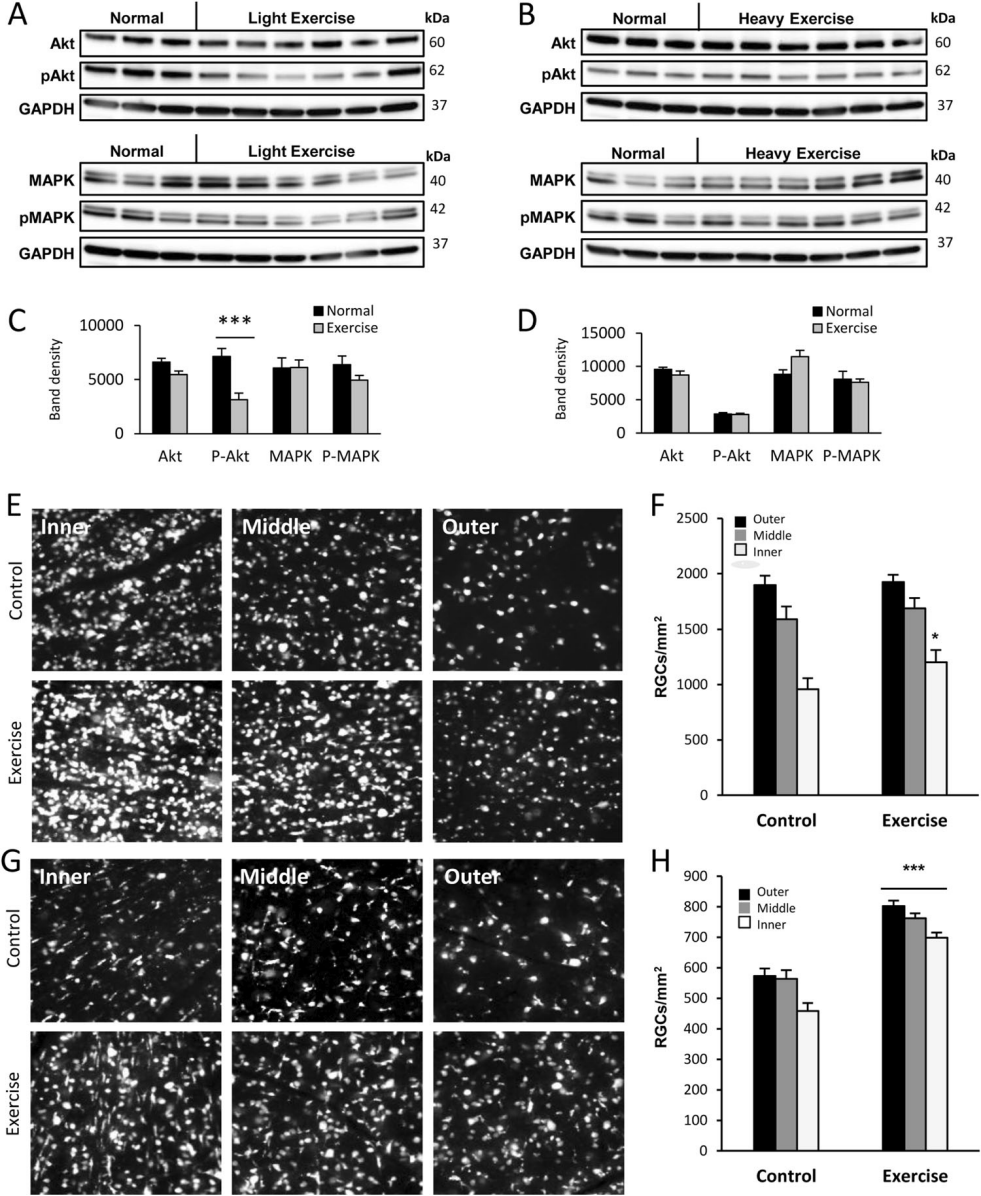
Exercise promotes RGC survival after optic nerve transection. **a**, **b** Western immunoblots of whole retinal lysates of control sedentary rats (Normal) and rats that underwent a forced light or heavy exercise program (exercise). Both groups were probed for active (phosphorylated; p-Akt or p-MAPK) and total Akt and MAPK. GAPDH loading controls are shown below each group of blots. **c**, **d** Graph depicting the mean normalized densitometry values (±SEM) of each group of blots for both treatments. **c** A forced light-exercise program caused a significant decrease in activated Akt levels (***p < 0.001), without significantly changing the levels of total Akt. **d** A forced heavy-exercise regimen did not significantly change the levels of Akt or MAPK, nor alter their activation in the normal adult retina (normal and exercise, *n* = 6 each). **e**, **h** Epifluorescence micrographs of flat-mounted retinas showing Fluorogold-labeled RGCs at 7 and 14 days after optic nerve transection in forced light or heavy exercise, respectively. The control retinas (*n* = 6, each) had few surviving RGCs. **e**, **g** Forced light- or heavy-exercise group (*n* = 6; each) increased RGC survival after optic nerve transection. **f** Forced light exercise partially increases the density of surviving RGCs at 7 days after injury (*p* < 0.05). **h** Forced heavy exercise enhances the density of surviving RGCs at 14 days after injury (*p* < 0.001). Scale bar, 50 μm

We initially examined whether forced exercise could modulate levels of active Akt (p-Akt) (Fig. 1a–d) and found that retinal p-Akt was significantly lower (*p* < 0.001) in lightly exercised animals relative to sedentary animals (Fig. 1a, c); however, levels of total Akt were unchanged, suggesting that exercise induced Akt dephosphorylation as opposed to reducing its expression. In contrast, levels of retinal p-MAPK and MAPK were unchanged (Fig. 1a, c), suggesting that exercise does not affect MAPK activation.

We next examined the effects of a heavy exercise regimen on PI3K and MAPK activation and expression levels. We found that heavily exercised animals did not show any significant differences (Fig. 1b, d). These findings suggest that heavy exercise does not affect the activation of the PI3K or MAPK pathways.

### Forced treadmill running improves the survival of adult CNS neurons

Optic nerve axotomy was used to study the effects of exercise on adult CNS neuron survival. Animals participated in a heavy exercise regimen because this program did not reduce the activation of the protective MAPK or PI3K/Akt pathways. Forced treadmill running prior to optic nerve axotomy improved RGC survival in the outer retinal eccentricity (Fig. 1e, f) at 7 days. The neuroprotective effect of exercise was more dramatic at 14 days post axotomy: RGC survival improved in all three eccentricities relative to controls (*p* < 0.001) (Fig. 1g, h). These findings demonstrate that exercise protects adult RGCs from apoptosis resulting from traumatic injury to the optic nerve.

### Cortical neuron survival after glutamate delivery is enhanced by a PTEN peptide

**Fig. 2:**
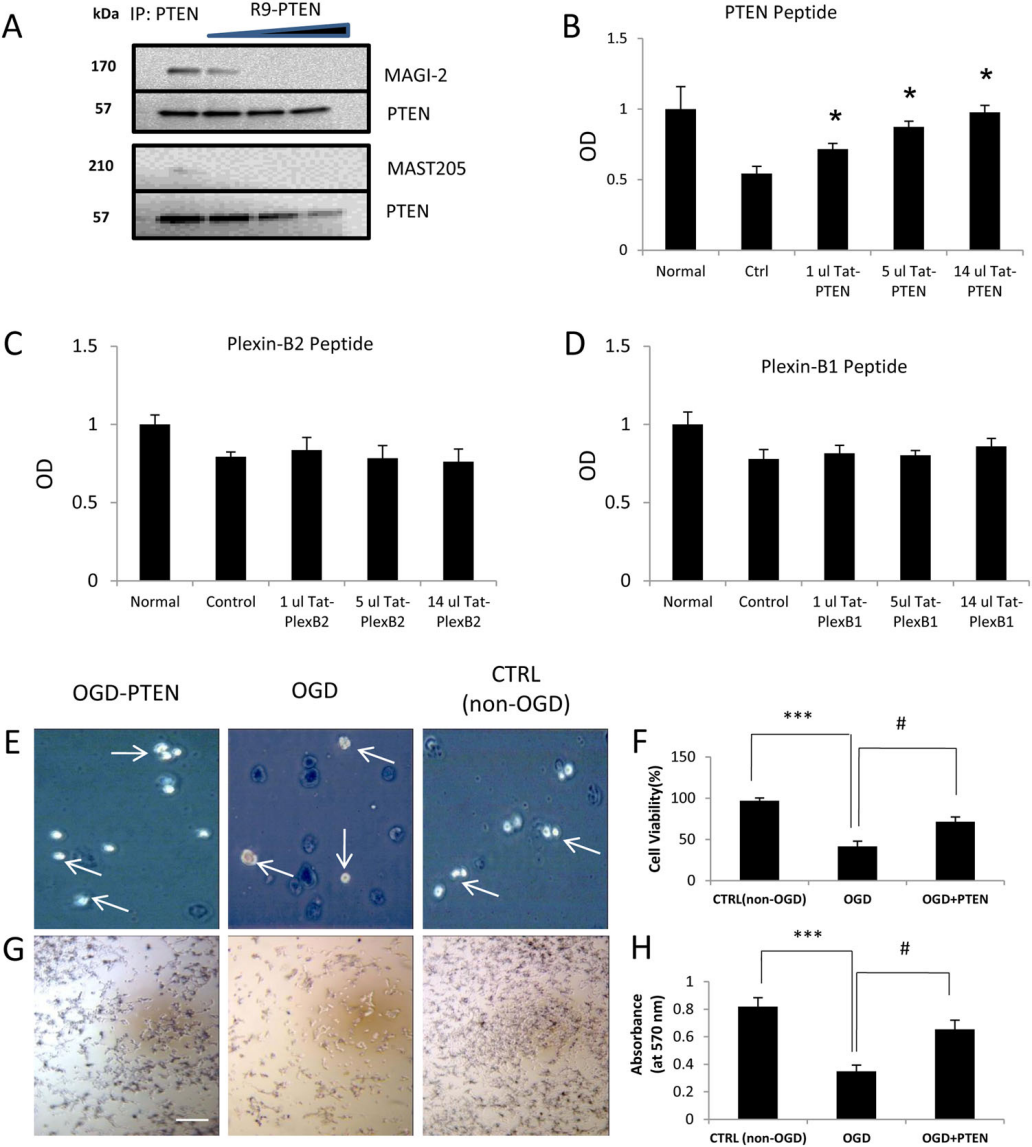
PTEN, which contains a PDZ-binding motif, interacts with MAST205 and MAGI-2 to induce cell death. **a** Immunoprecipitation was used to isolate PTEN from retinal lysate and assess its binding partners. Increasing concentrations of R9-conjugated PTEN peptide reduced the co-precipitation of MAGI-2 and Mast205, two known PTEN-binding partners. **b**–**d** Postnatal cortical neurons were cultured for 2 weeks to allow neurite development. Cells were killed by adding 5 mM glutamate to their medium for 24 h. Controls also received vehicle in the medium, whereas treatment groups also received increasing volumes of PTEN, Plexin B2, and Plexin B1 peptides. Survival is expressed relative to normal cells that did not receive glutamate (normal). The vertical axis represents the mean optical density (OD) (±SEM) relative to normal, untreated cells. Higher optical densities indicate greater cell numbers. **b** TAT-PTEN increased cell survival at 24 h, whereas **c** TAT-Plexin B2 or **d** TAT-Plexin B1 peptides did not. **e**–**h** HEK293 cells are submitted to OGD (3 h). One day after OGD; viability of the cells was quantified by **e**–**f** trypan blue extrusion test or **g**, **h** mitochondrial activity assay. Treatments that prevent PTEN interaction with the PDZ domain containing protein significantly rescued OGD-induced cell death. Data are average ± SEM (*n* = 3 independent experiments). *p < 0.05 relative to control, *p < 0.001 between control and the OGD group, ^#^*p* < 0.001 between OGD + PTEN and the OGD group, scale bar, 50 μm

To initially test the effectiveness of the PTEN peptide in preventing PTEN binding with known PDZ partners, an immunoprecipitation assay was used to isolate PTEN from retinal lysates and assess the co-precipitation of PDZ proteins. Increasing concentrations of R9-conjugated PTEN peptide reduced the co-precipitation of MAGI-2 and Mast205, two known PTEN-binding partners, revealing that the PTEN C-terminal peptide blocks PTEN binding to PDZ-scaffolding proteins (Fig. 2a).

The neuroprotective properties of the PTEN peptide were tested in vitro to determine whether peptide application could reduce neonatal cortical neuron degeneration in a glutamate toxicity assay. Increasing doses of the TAT-PTEN peptide improved cortical neuron survival at 24 h after glutamate challenge (Fig. 2b).

To examine the mechanism whereby PTEN improves neuron survival, peptides targeting Plexin B1 or B2 were also tested. Plexin B1 has been reported to activate PTEN leading to growth cone collapse^61^. In contrast to the results obtained following treatment with TAT-PTEN, neither TAT-Plexin B1 nor TAT-Plexin B2 human peptides affected cortical neuron survival after glutamate insult (Fig. 2c, d).

### PTEN PDZ-motif peptide promotes survival after OGD in vitro

The protective potential of the PTEN PDZ-domain peptide against ischemic cell death was assessed by exposing HEK293 cells to OGD, 3 h, followed by reperfusion (Fig. 2e–h). Pretreatment of HEK293 cells with R9-PTEN- (20 µM) enriched media for 2 h improved cell viability ~47% (from 41.4 ± 6.36 to 71.7 ± 5.42) in the trypan blue assay (Fig. 2e, g). R9-PTEN also increased cell survival by ~53% (from 0.35 ± 0.04 to 0.65 ± 0.07) relative to control (Fig. 2f, h). This shows that inhibition of PTEN PDZ interactions protects against OGD in vitro.

### PTEN peptide prevents neurodegeneration in vivo

**Fig. 3:**
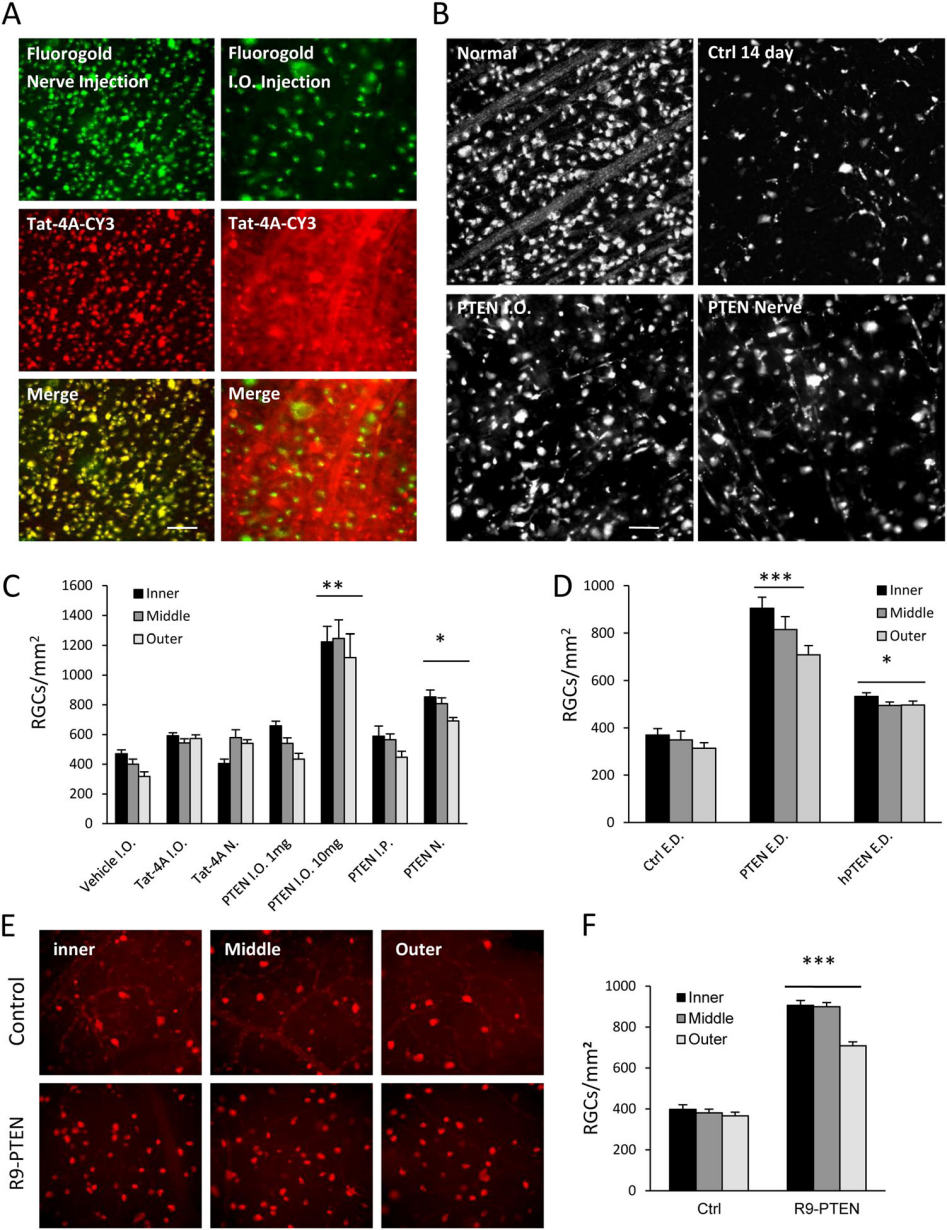
Tat-conjugated peptide promotes RGC survival after traumatic insult. **a** Quantification of the density (cells/mm^2^) of surviving RGCs( ± SEM) at 14 days after optic nerve transection. Epifluorescence images showing the cellular localization of Cy3-labeled Tat-conjugated peptide (Tat-P-CY3) following injection into the optic nerve (images on left) or intraocular (IO) injection (images on right). Nerve injection resulted in the selective transfection of axotomized RGCs that were retrogradely labeled with Fluorogold from the superior colliculus (colocalization shown in merged image at bottom; yellow). IO injection resulted in the widespread transfection of the inner retina as evidenced by labeling of RGCs, surrounding glia, and other neurons in the ganglion cell layer (red). RGCs (green) were pre-labeled with Fluorogold prior to IO injection of Cy3-labeled peptide. **b** Epifluorescence micrographs of flat-mounted retinas showing RBPMS-labeled RGCs at 14 days after optic nerve transection with PTEN (R9-PTEN) peptide administered via IO injection. Images were taken in the inner, mid-periphery (middle), or periphery (outer) of the retina. Treatment with PTEN peptide significantly increased RGC survival (***p < 0.001), Scale bar, 50 μm. *n* = 7 each group. Fluorescence images of Flourogold retrogradely labeled RGCs in a normal at 14 days post axotomy. **c** Quantification of RGCs shows that IO or nerve (N) injection and **d** eye drops (ED) of TAT-conjugated peptides prevented neuronal death. **e** Epifluorescence micrographs of flat-mounted retinas showing RNA-binding protein with multiple splicing (RBPMS)-labeled RGCs at 14 days after optic nerve transection with PTEN (R9-PTEN) peptide, IO injection, treatment. Images were taken in the inner, mid-periphery (middle), or periphery (outer) of the retina. **f** Quantification of the density (cells/mm^2^) of surviving RGCs( ± SEM) at 14 days after optic nerve transection. Treatment with PTEN peptide significantly increased RGC survival. (*p < 0.05, **p < 0.01,***p < 0.001), Scale bar, 50 μm. *n* = 7 each group

We next sought to determine if the PTEN PDZ peptide would prevent CNS neurodegeneration in vivo (Fig. 3a–d). TAT-PTEN was administered following injury via IP injection, IO injection, or injection into the optic nerve (N). Controls were TAT-conjugated polyalanine peptides (TAT-4A) injected via the same routes. There was no change in RGC survival between control-treated and axotomized untreated animals. IO injection of TAT-PTEN [10 mg/mL] produced a robust increase in RGC survival at 14 days post axotomy (Fig. 3b, c). A single nerve injection of TAT-PTEN (10 mg/ml) also enhanced RGC survival by ~2-fold (Fig. 3b, c). These results show that TAT-PTEN is an effective anti-apoptotic therapeutic for injured RGCs, and that additional benefits are derived from globally targeting the retina via IO injections.

### PTEN C-terminal peptide prevents neurodegeneration in vivo via eye drops

Eye drops are a clinically useful method of noninvasive therapy delivery to the injured retina^62^. We therefore examined the effect of eye-drop-delivered R9-PTEN peptide on RGC survival: treatment was administered once per day for the first 7 days after axotomy and increased RGC survival at 14 days (Fig. 3d). A peptide designed to target human PTEN, which differs from Rat PTEN by one amino acid residue, also RGC survival when administered via eye drops (Fig. 3d). Together, these findings show that targeting the PTEN PDZ motif has therapeutic potential for preventing RGC degeneration when delivered via eye drops.

### R9-protein transduction sequence has a similar effect to TAT-protein transduction domain

To contrast the efficacy of different protein transduction sequences in vivo, we evaluated the effects of intraocularly-injected R9-conjugated PTEN peptide after axotomy and found that average density in the R9-PTEN peptide group was significantly increased to 616.63 ± 21.41 cells/mm^2^ (*p* < 0.001; Fig. 3e, f). Overall, the R9-conjugated PTEN peptide showed similar neuroprotective effects to TAT-conjugated PTEN peptide.

### Effects of PTEN C-terminal peptide after ischemia in the mammalian retina

**Fig. 4:**
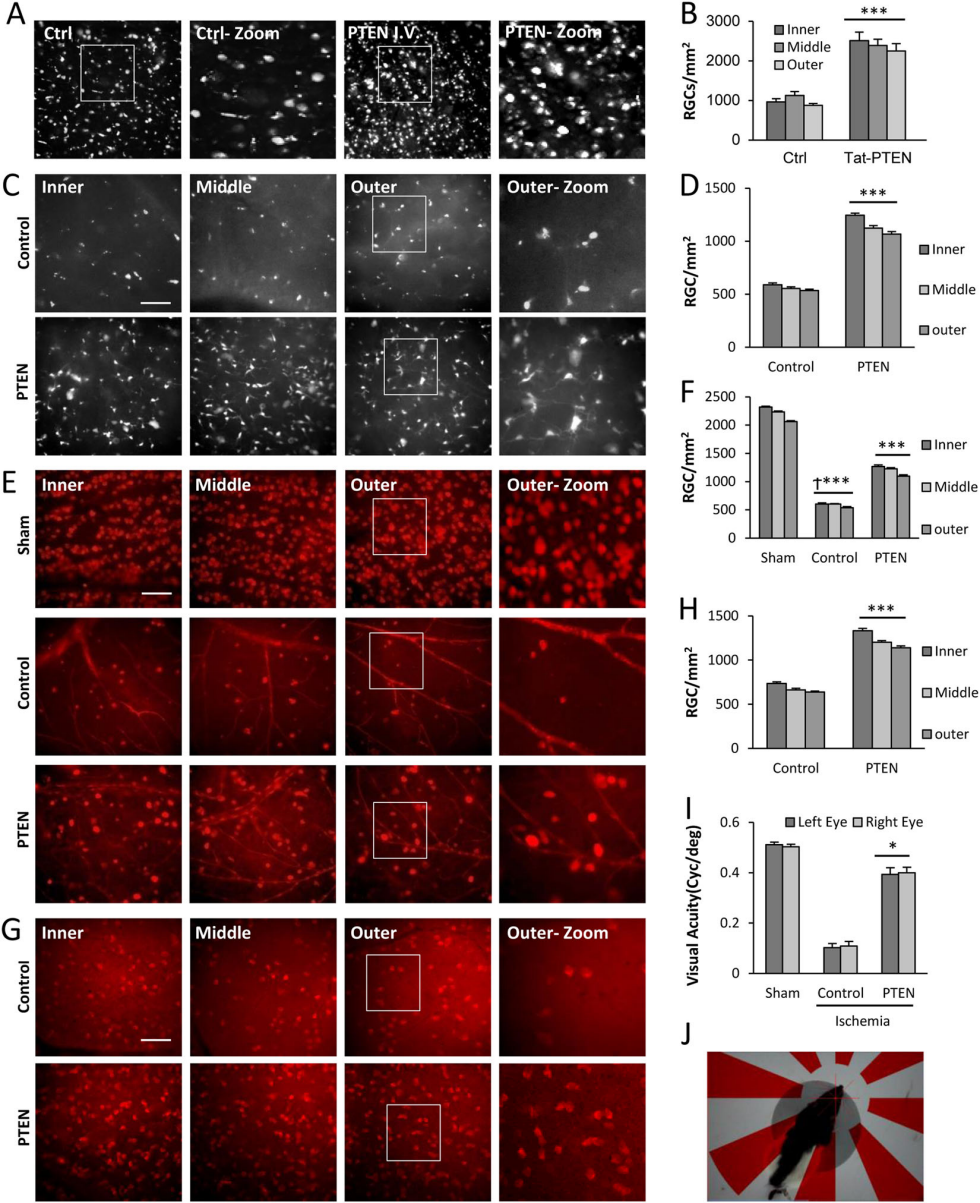
PTEN peptide promotes RGC survival after ischemia. **a** Epifluorescence micrographs of flat-mounted retinas showing Fluorogold-labeled RGCs at 14 days following ophthalmic artery ligation and Tat-PTEN peptide, intravenous(I.V.) injection, treatment; CTRL(control) retinas (*n* = 6) had few surviving RGCs; Tat-PTEN (*n* = 6) increased RGC survival after retinal ischemia. **b** Quantification of the density (cells/mm^2^) of surviving RGCs (±SEM) at 7 days following ophthalmic artery ligation and IV treatment with Tat-PTEN peptide. Tat-PTEN peptide significantly increased RGC survival (***p < 0.001) after retinal ischemia. **c** Epifluorescence micrographs of flat-mounted retinas showing Fluorogold retrograde lableled RGCs at 14 days after retinal ischemia. **d** Retinas that received IO injections of R9-PTEN had significantly higher RGC densities than controls. **e** Epifluorescence micrographs of RBPMS immunolabeled RGCs at 14 days after ischemia. **f** R9-PTEN treated retinas had significantly higher RGC densities than controls, approximately half of sham treatment. **g** Epifluorescence micrographs showing RBPMS immunolabeled RGCs at 7 days after IO pressure elevation. **h** IO R9-PTEN treatment significantly enhanced RGC survival after elevated IOP. All RGC density quantification was express and the mean number of cells/mm^2^ (±SEM). *** = *p* < 0.001. **i** The optomotor responses after IOP elevation was quantified. Visual acuity increased from 0.11 cpd in the control (ischemia + vehicle) group to 0.40 cycle per degree (cpd) in the PTEN (ischemia + PTEN peptide) group whereas it was 0.51 cpd in sham (no ischemia) group. **j** Photograph showing the Long–Evans rat in the rotating drum under phototopic condition. PTEN peptide significantly improved visual performance after retinal ischemia. (*p < 0.05, ***p < 0.001 relative to control, and Ϯ*p* < 0.001 relative to sham), Scale bar, 50 μm. *n* = 7 each group

Intravenous injection of TAT-PTEN resulted in a several-fold increase in RGC survival at 7 days post ischemia (Fig. 4a, b). IO injection of R9-PTEN significantly increased RGC survival several-fold relative to control, whether RGCs were identified by retrograde labeling with Fluorogold (Fig. 4c, d), or immunohistochemistry for RBPMS (Fig. 4e, f). Together, these results demonstrate the ability of cell-permeable PTEN PDZ-domain targeting peptides to prevent the degeneration of RGCs after ischemia.

Finally, we tested the effects of PTEN peptides in a model of elevated IO pressure (IOP). At 7 days after acute IOP elevation, control eyes showed a mean RGC density of 677.5 ± 16.25cells/mm^2^ (Fig. 4g, h) while those treated with R9-PTEN showed a 44% increase (*p* < 0.001; Fig. 4h) in RGC survival (1223.17 ± 22.92 cells/ mm^2^). Together, these results show that the PTEN PDZ-motif peptide significantly protects RGCs from cell death induced by high IOP.

To examine functional recovery after elevated IOP, optomotor responses were quantified at 7 days after injury (Fig. 4i, j). Visual acuity increased from 0.11 cpd in the control (ischemia + vehicle) group to 0.40 cpd in the PTEN (ischemia + PTEN peptide) group, whereas it was 0.51 cpd in the sham (no ischemia) group. These results show that R9-PTEN peptide significantly improves functional visual acuity after elevation of IO pressure.

**Fig. 5:**
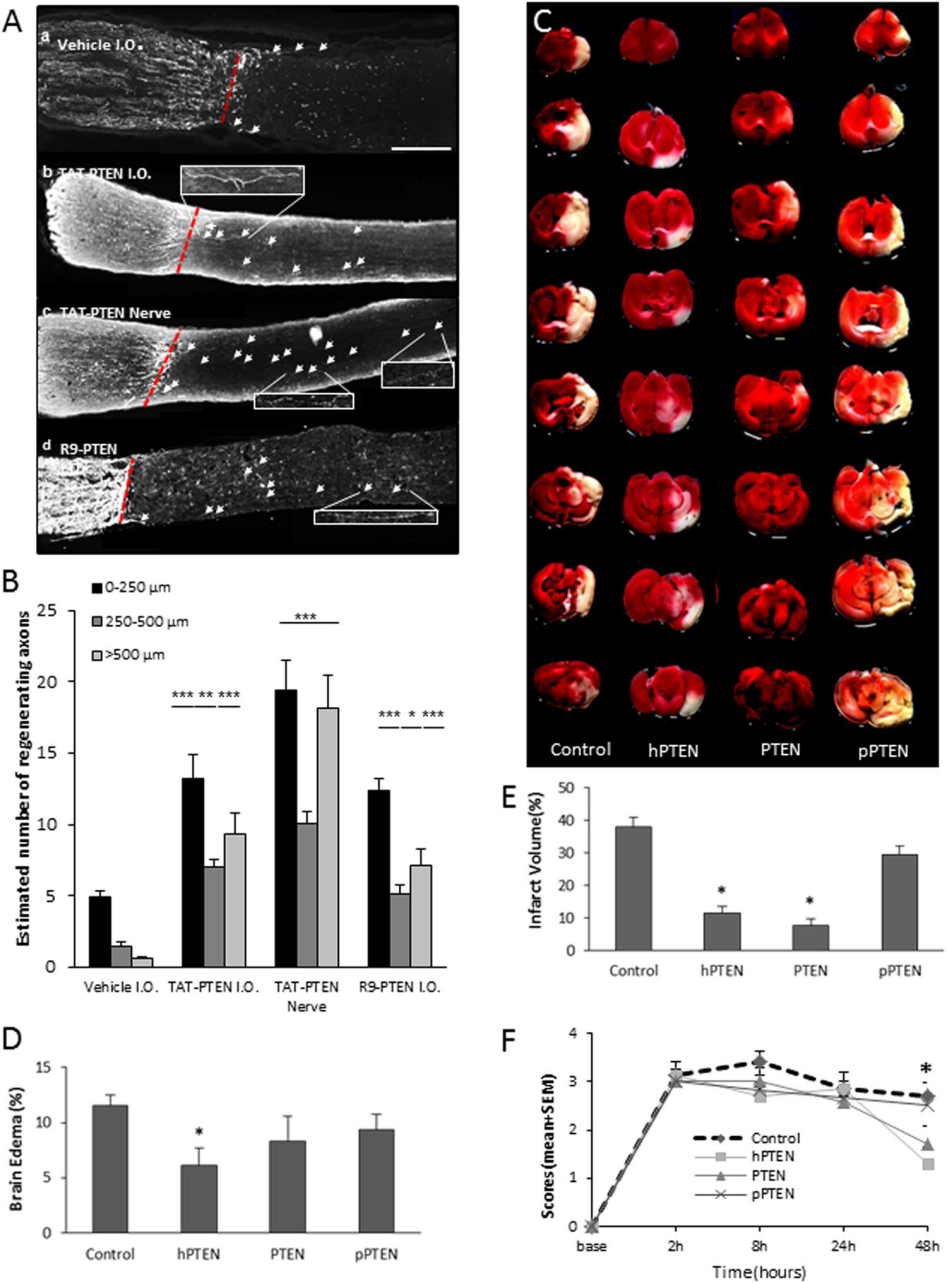
PDZ peptides that target PTEN enhance axonal regeneration and reduced ischemic brain injury after stroke. **a**_a–d_ Epifluorescence micrographs of GAP-43-immunolabelled sections of optic nerves fixed at 21 days after optic nerve crush. IO injections were delivered at 3- and 10 days following injury; TAT-PTEN-soaked gelfoam (**a**_c_) was applied immediately to the lesion site after crush. Crush site is indicated by arrowhead. Arrows demarcate some of the regenerating axons in each section. The retina (not visible) is towards the left-hand side of each image. Bar = 250 μm. **a**_a_ Nerve that received vehicle IO treatment showed minimal axonal regeneration beyond the crush site. **a**_b_, **a**_c_ Nerves treated with the TAT-PTEN peptide either by IO delivery (**a**_b_) or via gelfoam application (**a**_c_) showed enhanced axonal regeneration into the distal optic nerve. **a**_d_ IO R9-PTEN-treated nerve showed similar enhancement of axonal regeneration. **b** Quantification of the mean number of regenerating axons (±SEM) at 21 days following optic nerve crush. The number of axons was quantified in three bins distal to the crush site (0–250 μm, 250–500 μm, and >500 μm). IO injections (I.O.) of TAT-PTEN or R9-PTEN significantly increased RGC axon regeneration compared to vehicle controls in the corresponding bin. The greatest number of regenerating axons was observed in the nerves that received the TAT-PTEN peptide applied directly at the lesion site (nerve). **c** Coronal brain slices showing the representative infarct area in a sample from control, hPTEN, PTEN (R9-PTEN), or pPTEN treated brains. Brains were processed by TTC staining to detect the infarcted brain region (white area) within the remaining metabolically active brain tissue (red). **d** Effect of Control, hPTEN, PTEN (R9-PTEN) or pPTEN peptides, intravenous injection, treatment on infarct volume (percentage of unlesioned hemisphere). **e** Effect of PDZ peptides against PTEN on brain edema percentage relative to the uninjured hemisphere. **f** Neurological deficits after stroke were assessed using the Bederson Scoring System. Graph depicts the Bederson scores for each of the four experimental groups: control (Ctrl), hPTEN, PTEN (R9-PTEN), and pPTEN. Bederson scores were evaluated at baseline, 2, 8, 24, and 48 h after MCAO. The neurological scores improved in both treated (PTEN and hPTEN) groups at 2 days after embolization. Ipsilateral assumed as ischemic and contralateral assumed as nonischemic hemisphere. Data are presented as mean ± SEM (***p < 0.001; **p < 0.01; *p < 0.05; vehicle I.O.; TAT-PTEN I.O.; TAT-PTEN gelfoam; R9-PTEN I.O.), *n* = 7 each group, Scale bar, 200 μm

RGC axon regeneration can be enhanced using a PDZ peptide directed against PTEN has been localized to the growth cones of budding axons^63^, and may be involved in the signaling of several neurite outgrowth inhibitors^63,64^. Accordingly, TAT-PTEN peptide was administered to the lesion site. Treatment with vehicle showed axonal regeneration past the crush site, whereas IO injections of TAT-PTEN (Fig. 5a) showed a significantly greater number of regenerating growth cones in all three bins (***p < 0.001 in the 0–250- and >500-μm bins; **p < 0.01 in the 250–500-μm bin) (Fig. 5b). Similarly, treatment with R9-PTEN showed a significantly greater number of regenerating growth cones in all three bins (***p < 0.001 in the 0–250- and >500-μm bins; *p < 0.05 in the 250–500-μm bin). Axonal regeneration was maximal in treatment with TAT-PTEN via gelfoam (Fig. 5a, b; ***p < 0.001). These results demonstrate that the TAT-PTEN and R9-PTEN peptides are capable of enhancing RGC axon regeneration following injury to the optic nerve.

### PTEN peptides reduce brain ischemic injury after MCAO

Intravenous delivery of R9-conjugated human PTEN peptide (hPTEN), or rat PTEN peptide (PTEN), significantly reduced infarct volume relative to control at 48 h after MCA occlusion (Fig. 5c, d). Compared with the control group (38 ± 3.01), infarct volume was reduced by 71 and 79% in the ischemic rats that received hPTEN (11.65 ± 1.9) or PTEN (7.76 ± 2.05), respectively (*p* < 0.001; Fig. 5d). Brain edema was significantly reduced following intravenous delivery of R9-hPTEN, but not rat PTEN or pPTEN, at 48 h after MCA occlusion (Fig. 5e).

At 2 and 8 h after MCAO, all animals showed significant motor deficits, with median scores of 3 for all groups. At 24 and 48 h after MCA occlusion, neurological scores were significantly improved by PTEN peptide treatment (both *p* < 0.05), whereas hPTEN only showed a difference at 48 h after injury (Fig. 5f). This reveals that PTEN or hPTEN peptides produce a delayed behavioral improvement after stroke; however, phosphorylation of the PTEN peptide PDZ domain abolishes this protective effect.

Seizures were observed in four rats in the control group at 2, 8, or 24 h after embolization. In contrast, one animal in the PTEN-treated group showed seizures at 2 and 8 h after ischemia. One animal in the hPTEN peptide treated group also showed seizures at 2, 8, and 24 h after embolization. Three animals in the pPTEN group showed seizures at 2, 8, and 24 h after ischemic injury. The overall mortality rate within these groups was 10.7% (3 out of 28) between 2 and 8 h after stroke.

## Discussion

Novel pharmacological approaches to reduce apoptosis and improve recovery in the injured CNS would meet an enormous healthcare need^65^. The present study evaluated the potential for modulation of the PI3K or MAPK pathways, through exercise or via peptide targeting, to increase survival or regeneration of RGCs and cortical neurons following CNS injury. Regarding the mechanism of action, our findings show that TAT-PTEN or R9-Conjugated PTEN peptides reduced the co-precipitation of MAGI-2 and Mast205, demonstrating that the PTEN peptide blocked PTEN binding to these scaffolding proteins. Taken together, these results demonstrate that the PDZ-blocking peptides that were developed in this study are potential treatments for CNS insults or diseases where neurons undergo apoptotic cell death.

Animals that underwent a 2-week exercise program showed decreased RGC apoptosis following optic nerve transection. These results agree with findings in d-Galactose-induced aged rats^29^. Together with our results, it is evident that exercise has a significant effect on reducing CNS degeneration. Previous findings showed that the levels of neurotrophins in the CNS were increased in response to exercise^25,66^. BDNF has been implicated in these protective effects and is known to activate both the PI3K and MAPK pathways in the retina^28^; however, we observed no significant increases in PI3K or MAPK phosphorylation between heavily exercised and sedentary animals. In contrast, the light exercise group showed a decrease in the levels of phosphorylated Akt. These results contradict previous findings in animal models of age and disease^29,67^. It appears that in the retina, the neuroprotective mechanisms of forced exercise are independent of these previously described phenomena, and possibly differ from voluntary exercise paradigms. It is also possible that exercise may enhance PI3K and MAPK activation in aged or diseased conditions, but not under normal healthy conditions. This may explain why the retinas from the animals that underwent a light exercise program showed a decreased level of phosphorylated Akt. Nevertheless, exercise was able to increase RGC survival after injury, which suggests that additional mechanisms contribute to this effect aside from the previously described PI3K/MAPK activation. Accordingly, the mechanisms by which exercise exerts its benefits in the CNS warrant further investigation.

The PI3K and MAPK pathways are essential in preventing apoptosis in injured RGCs^28^; however, these beneficial effects are restricted by negative regulators such as PTEN, Erbin, and BCR. While this is important in healthy adult cells, these negative regulators become obstacles for the survival of adult neurons upon ischemic or traumatic CNS injury. We utilized small peptides mimicking the PDZ-binding motif of PTEN, to disrupt PTEN-binding interactions^31^. Typically, a PDZ interaction involves a PDZ-binding motif and the binding pocket with which it interacts, called a PDZ domain^30,31^. PI3K is activated by receptor tyrosine-kinases, upon induction by ligands^68^. Activated PI3K phosphorylates phosphatidylinositol-4,5-bisphosphate (PI-4,5-P_2_) producing phosphatidylinositol-3,4,5-trisphosphate (PIP_3_)^69^. PIP_3_ second messengers activate protein kinase B (Akt)^70^, which is involved in numerous processes, including cell cycle progression, cell growth, cell differentiation, cell survival, and anti-apoptotic functions^71^. PTEN hydrolyses PIP_3_ to PI-4,5-P_2_, reducing the number of second messengers that activate PKB/Akt, and thus, negatively regulates the PI3K pathway^2^. PTEN contains a PDZ-binding motif, through which it interacts with hDLG, MAST205, and MAGI-2^72^. We disrupted the PDZ binding of PTEN through application of a small molecular inhibitor. PTEN PDZ-motif peptides decreased apoptosis in both in vitro and in vivo injury models. The likely mechanism for this effect is reduced PTEN recruitment and stabilization at the plasma membrane. This is supported by previous findings in the human hepatocarcinoma 7721 cell line, where MAGI-2 was found to inhibit cell migration and proliferation in a PTEN-dependent manner^20,21^.

The effect of TAT-PTEN and R9-PTEN on RGC axon regeneration was also evaluated in the present study. PTEN is necessary in the signaling of several neurite outgrowth inhibitors, including Sema3A, Sema4D, and MAG^63,64^, which accumulate at lesion sites in the CNS and prevent axonal regeneration^5,6^. PTEN therefore represents a common entry point onto which these molecules converge and transmit their growth-inhibiting signals. This study found that TAT-PTEN enhanced axonal regeneration in the optic nerve: animals treated with either TAT- PTEN or R9-PTEN showed a significant augmentation in the number of regenerating growth cones beyond the injury site following optic nerve crush. These results agree with recent findings in which the reduction of PTEN, either through genetic deletion or shRNAs, increased corticospinal tract sprouting and axon regeneration following injury in the optic nerve and corticospinal tract^73^. Thus, our results indicate that peptides for disrupting PTEN binding represent a potential therapeutic approach for the treatment of both CNS degenerative diseases and traumatic axonal injury.

Glaucoma is a leading cause of acquired blindness, which may involve an ischemia-like insult to RGCs and the optomotor response^56^. We demonstrated that visual acuity increased fourfold to 0.40 cpd in the PTEN treated groups relative to control. Thus, modifying PTEN recruitment by PDZ-blocking peptides also improves RGC survival and functional recovery following elevated IOP, having potential therapeutic applicability in Glaucoma.

PTEN inhibition has been reported to be neuroprotective in experimental models of cerebral ischemia/reperfusion injury; however, PTEN deletion has also been shown to lead to cognitive impairment^74^. During ischemia/reperfusion injury, PTEN activity can be regulated by several post-translational modifications^75–77^. Beneficial actions of PTEN inhibition are usually attributed to upregulation of PI3K and downstream signaling through Akt^78^. Furthermore, downregulating of PTEN expression has been to shown to inhibit extra synaptic NMDA receptor activity, and thus attenuate excitotoxicity induced by cerebral ischemia^79^. The results of the present study show that treatment with rat or human PTEN peptides was significantly neuroprotective following embolic stroke in rats: systemic administration of PTEN peptides immediately following and after ischemic injury significantly reduced infarct volume by ~71%. Rats that received PTEN peptides also demonstrated improved functional recovery and reduced neurological deficits. Taken together, these results clearly demonstrate that the PDZ-motif peptide is an effective treatment for ischemic brain injury.

## Conclusions

Exercise induced minor alterations in the activation of the PI3K and MAPK pathways, leading to a significant increase in RGC survival after injury. These findings corroborated previously published work and suggested an important role for these molecules in CNS regeneration. In addition, targeting negative regulators of the PI3K and MAPK pathways produced a robust enhancement in the survival and regeneration of RGCs; most importantly, visual acuity in the injured retina was also improved, cerebral infarct volume was decreased, and neurological deficits were improved. As such, the PDZ-blocking peptides developed in this study have potential therapeutic value in treating CNS insults or diseases where neurons undergo apoptotic cell death.
